# Characterizing miRNA editing patterns in 5 types of cells using single-cell small RNA sequencing data

**DOI:** 10.3389/fbinf.2026.1719535

**Published:** 2026-04-09

**Authors:** Chunyi Mao, Hao Guo, Wenping Xie, Yue Xu, Hongjia Zhang, Kang Luo, Jun Yang, Yun Zheng

**Affiliations:** 1 College of Big Data, Yunnan Agricultural University, Kunming, Yunnan, China; 2 College of Horticulture and Landscape, Yunnan Agricultural University, Kunming, Yunnan, China; 3 Department of Cardiology, The First Affiliated Hospital of Kunming Medical University, Kunming, Yunnan, China; 4 School of Criminal Investigation, Yunnan Police College, Kunming, Yunnan, China

**Keywords:** glioblastoma, hsa-mir-376c_48_A_g, MicroRNA (miRNA), miRNA editing, single cell

## Abstract

Numerous studies have identified a large number of miRNA editing sites via deep sRNA sequencing profiling of tissue samples. However, the single-cell landscape of miRNA editing patterns has remained largely unknown to date. To investigate miRNA editing and mutation characteristics at single cell level, this study analyzed miRNA editing and mutation events in 448 single-cell small RNA sequencing profiles from 5 different cell types. Our results revealed that PCA and clustering analysis, performed based on the editing levels of identified miRNA editing sites, could distinguish distinct cell types, indicating that miRNA editing patterns are cell-type-specific across different cellular populations. We further demonstrated that a subset of miRNA editing sites exhibited strict cell-type-specific editing patterns. Meanwhile, within the same cell type, the identified sites presented different distributions of editing levels in different cells. A fraction of sites showed highly variable editing levels among different cells of the same cell type, while some sites displayed relatively uniform and consistent editing patterns. An A-to-I editing site in hsa-mir-376c, i.e., hsa-mir-376c 48 A g, showed a significantly higher editing level in glioblastoma cells than in naive embryonic stem cells, suggesting a potential role in the initiation and progression of glioblastoma. Furthermore, our results also suggest that in leukemia cells, TENT4A, TENT5A, TENT5B, TENT5C, TENT5D, and TUT1 may mediate the non-templated nucleotide additions to the 3′ends of miRNAs.

## 1 Introduction

MicroRNAs (miRNAs) are a class of single-stranded non-coding RNAs of approximately 22 nucleotides (nt) in length (Ambros, 2001), which negatively regulate gene expression at the post-transcriptional level (Hutvagner and Zamore, 2002; Agarwal et al., 2015; Lu and Rothenberg, 2018; Gan and Gunsalus, 2019). The primary transcripts of miRNAs (pri-miRNAs), which bear a 5′ cap and a 3′ poly(A) tail, are cleaved by the RNase III family nuclease Drosha to generate miRNA precursors (pre-miRNAs) (Lee et al., 2004; Denli et al., 2004). Pre-miRNAs are single-stranded RNAs with hairpin structure approximately 70 nt (Denli et al., 2004). Pre-miRNAs are exported from the nucleus to thecytoplasm by the nuclear export receptor, Exportin-5 (Yi et al., 2003). In the cytoplasm, Dicer catalyzes the processing of pre-miRNAs, consequently generating mature miRNA duplexes (Hutvagner et al., 2001; Ketting et al., 2001).

The miRNA duplexes assemble with Argonaute (*AGO*) family proteins to form the RNA-induced silencing complex (RISC) (Kobayashi and Tomari, 2016). Of the two strands, one is selected to function as the mature miRNA, while the other is degraded under physiological conditions (Krol et al., 2010; Kobayashi and Tomari, 2016). As key post-transcriptional regulators, miRNAs are involved in a wide range of fundamental cellular processes, including cell growth, differentiation, development and apoptosis (Lee and Calin, 2011; Lin et al., 2019; Saliminejad et al., 2019; Hu et al., 2020; Liu et al., 2020). As part of the RISC, miRNAs base-pair with the 3′ untranslated regions (3′-UTRs) of target mRNAs, and trigger translational repression or degradation of the target transcripts (Bartel, 2009; Fabian et al., 2010). Accordingly, numerous computational and experimental methods have been developed for the prediction and identification of miRNA target genes (Enright et al., 2003; Krek et al., 2005; Chi et al., 2009; Hafner et al., 2010; Zheng and Zhang, 2010; Agarwal et al., 2015; Zheng, 2018).

RNA editing is an important post-transcriptional regulatory mechanism that significantly contributes to the diversity of RNA and protein expression (Zinshteyn and Nishikura, 2009; Nishikura, 2010; Xu et al., 2021). During the physiological process of miRNA molecules maturing from primary transcripts, their sequences can also undergo various forms of editing (Zheng, 2018). A-to-I editing of RNA is catalyzed by *ADAR* proteins, which are conserved in mammals (Nishikura, 2016; Marceca et al., 2021; Yuting et al., 2021). A-to-I editing of miRNAs is widely spread in human brains (Kawahara et al., 2008; Guo et al., 2022; Lu et al., 2022; Wu et al., 2023). C-to-U editing of RNA is mediated by *APOBEC* family members (Prochnow et al., 2009; Salter et al., 2016; Correia de Sousa et al., 2019). The 3′ non-templated nucleotides addition of RNA is generated by terminal nucleotidyltransferases (*TENTs*) (Yu and Kim, 2020). The maturation of miRNA is mediated by adenylation and uridylation, which represent the addition of an adenosine (A) or uridine (U) nucleotide to the 3′ end of miRNA transcripts, respectively (Yu and Kim, 2020; Kim et al., 2020). Among members of the *TENT* family, *TUT4* and *TUT7* exhibit genetic redundancy in the process of uridylation of mRNAs, and can selectively bind to mRNA targets with short poly(A) tails (generally less than 25 nt) during their action (Lim et al., 2014). *PABPC1* can bind to the 25-nt short poly(A) tails of mRNA molecules, thereby preventing *TUT4* and *TUT7* from binding to them (Lim et al., 2014). Therefore, uridylation catalyzed by *TUT4* and *TUT7* can only occur when the poly(A) tail is less than 25 nt and the last *PABPC1* protein dissociates from the 3′ end of the mRNA (Lim et al., 2014).

MiRNAs primarily rely on base pairing between their seed regions and complementary sites on target mRNAs to regulate gene expression (Bartel, 2009; Marceca et al., 2021). Therefore, any nucleotide change in the seed region may significantly alter the target genes of miRNAs (Kawahara et al., 2007; Marceca et al., 2021). The occurrence of editing events can disrupt the complementarity between miRNA and its targets, while introducing new targets (Kawahara et al., 2007; Marceca et al., 2021). Notably, miRNA editing events do not completely replace the original target genes, but instead partially introduce new ones (Zheng et al., 2016; Marceca et al., 2021).

Numerous studies have been conducted to elucidate the regulatory functions of edited miRNAs in a wide spectrum of complex diseases, largely based on bulk RNA sequencing (RNA-seq) data. For examples, Choudhury et al. suggested that the seed region of miR-376 clusters underwent A-to-I editing in human brains (Choudhury et al., 2012). The unedited and edited miR-376-3p could inhibit the expression of *RAP2A* and *AMFR*, respectively, to promote the metastasis and invasion of glioblastoma cells (Choudhury et al., 2012). Lu et al. demonstrated that miR-497-5p with A-to-I editing had significantly higher expression levels in PD tissues samples compared with normal samples (Lu et al., 2022). Furthermore, overexpression of the edited miR-497-5p could downregulate the expression of *OPA1* and *VAPB* potentially to inhibit the proliferation of glioma cells (Lu et al., 2022). Other studies further characterized the functional roles of miRNA editing, revealing that edited miRNAs can drive or suppress tumor progression (Shoshan et al., 2015; Xu et al., 2019; Qin et al., 2022), reverse cellular drug resistance (Romano et al., 2023), and modulate core pathological processes in neurodegenerative and neurodevelopmental disorders (Guo et al., 2022; Wu et al., 2023).

Only a few studies generated single-cell sRNA sequencing profiles due to technological difficulties. In 2016, Faridani et al. generated 429 single cell sRNA-Seq profiles from 8 types of cell lines (Faridani et al., 2016). Wang et al. performed simultaneous co-sequencing of miRNAs and mRNAs for 19 cells from a leukemia cell line (Wang N. et al., 2019).

A large number of edited miRNAs have been identified based on bulk RNA sequencing data, and their regulatory mechanisms involved in physiological activities have been revealed (Choudhury et al., 2012; Zheng et al., 2014; Shoshan et al., 2015; Zheng et al., 2016; Wang Q. et al., 2019; Xie et al., 2022; Guo et al., 2022; Qin et al., 2022; Lu et al., 2022; Romano et al., 2023; Wu et al., 2023; Liu et al., 2023; Guo et al., 2024). However, bulk RNA sequencing profiles represent the average level of cell populations and mask heterogeneity between different types of cells, particularly in tumor tissues. Therefore, it is essential to investigate miRNA editing events at single cell level, which may provide a new perspective for understanding intercellular heterogeneity. In this study, we analyzed the miRNA editing events in 448 single cell sRNA sequencing profiles from five types of cells. Our results indicate that different types of single cell have different miRNA editing patterns. Some editing sites show different editing levels in different types of cells and even in different single cells of the same cell types. These results provide a new view of miRNA editing at the single cell level.

## 2 Materials and Methods

### 2.1 Small RNA and RNA sequencing profiles used

To comprehensively identify miRNA mutation and editing (M/E) sites in single-cell sRNA sequencing samples, we collected 448 single-cell sRNA sequencing profiles from NCBI SRA database (Sayers et al., 2023) with their accession numbers in Supplementary Table S1. Firstly, 429 samples from 7 different cell lines were generated by Faridani et al. (2016). After combing cells from four glioblastoma cell lines (KS4, JM3, JM4 and U87), 165 glioblastoma (GBM) cell samples were obtained. Then, 66 HEK293FT cell samples, 95 primed ESC (pESC) samples, and 103 naive ESC (nESC) samples were also produced in (Faridani et al., 2016). Secondly, another study generated 19 cell samples (Leuk) from one leukemia cell line (K562) (Wang N. et al., 2019). To further investigate the relationships between miRNA editing events and their corresponding enzymes in single cells, we also downloaded RNA sequencing profiles of 19 Leuk cell samples (Wang N. et al., 2019). The qualities of the sRNA profiles were evaluated with FastQC program (Wingett and Andrews, 2018).

### 2.2 Genome sequence and miRNA annotation profiles used

The human unmasked genomic sequences (GRCh38) were downloaded from UCSC Genome Browser (Nassar et al., 2023). The index of human genomic sequences were generated with the bowtie-build program in the Bowtie package (Langmead and Salzberg, 2012). The sequences of pre-miRNAs and mature miRNAs, and genomic loci of miRNAs in GFF3 format were downloaded from the miRBase (release 21) (Kozomara and Griffiths-Jones, 2014).

### 2.3 Identifying microRNA editing and mutation sites in single cells

The 448 single-cell microRNA sequencing profiles were analyzed using the MiRME algorithm (Zheng et al., 2016; Zheng, 2018) with the default settings and parameters. Briefly, the first 25 nucleotides from 5′ end of raw reads had sequencing scores of 30 or higher were kept as qualified reads. Then, we obtained the unique sequences of the remaining reads and calculated the counts of unique reads with more than 18 nucleotides. Next, we aligned the unique reads to human pre-miRNAs using NCBI BLASTN (Altschul et al., 1990) with the options of “-S 1 -m 8 -e 0.01” and the reads mapped to human pre-miRNAs were retrieved. Next, these reads mapped to pre-miRNAs were aligned to the genome using Bowtie (v1.3.1) (Langmead et al., 2009) with the options of “-a -best -S -v 1”. Then, the alignments of reads to genome were examined by the cross-mapping correction method (De Hoon et al., 2010) to adjust the weights or percentages of a unique read at each of its genomic loci. In the main step, the MiRME algorithm with the default parameters was used to identify.

M/E sites in miRNAs, which was based on the sequences and structures of pre-miRNAs, the alignment of reads to the genome generated by Bowtie, the reads mapped to pre-miRNAs, the alignments of reads to pre-miRNAs generated by BLASTN, and the results of the cross-mapping correction method (De Hoon et al., 2010).

The following criteria were used to define editing sites with significant editing levels: (i) the relative editing level was at least 5%; (ii) at least 10 reads supported the editing event; (iii) the score threshold of sequencing reads was 30; and (iv) a multiple-test corrected P -value (using the Benjamini and Hochberg method (Benjamini and Hochberg, 1995)) was smaller than 0.05. Then, the obtained results of different samples were combined by a separate program in the MiRME package (see details in (Zheng et al., 2016; Zheng, 2018)). Based on the positions of M/E sites in miRNAs and mutations in dbSNP, the identified M/E sites were classified into nine different editing types, i.e., A-to-I, C-to-U, 3′-A, 3′-U, 3′-Other, 5′-editing, Other, SNP, and Pseudo. (Zheng et al., 2016) Only those sites that had significant editing levels in at least 5% (23 samples) of 448 samples were kept for further analysis (as listed in Supplementary Table S2).

We also examined the correlation between editing levels of miRNA M/E sites and their corresponding (or potential) enzymes for the 19 Leuk cells. Only those sites with significant editing levels in at least one of the 19 samples were retained for further analysis (as listed in Supplementary Table S7).

Each identified M/E sites was named according to the name of pre-miRNA, the location of the site, the nucleotides in the reference pre-miRNA sequence, and the edited/mutated nucleotides on the site. The original nucleotide in upper case and the edited/mutated nucleotied in lower case (Zheng et al., 2016; Zheng, 2018). The edited miRNA corresponding to a miRNA editing site was named according to the name of pre-miRNA, the location of the site, and the edited/mutated nucleotides on the site. For example, hsa-mir-376a-1_49_A_g is an A-to-I editing site on the 49th nucleotide of pre-miR-376a-1, and hsa-mir-376a_49 g is the edited hsa-miR-376a-3p corresponding to this editing site (Lu et al., 2022).

### 2.4 Comparing miRNA M/E sites to reported SNPs and editing sites

The sites satisfied the following criteria were defined as SNPs: (i) they shared identical genomic positions with the SNPs, (ii) they had the same nucleotides as the alleles of the SNPs for both the original and changed nucleotides, and (iii) they had editing levels of 100% in at least one of the samples selected.

We then compared the miRNA M/E sites identified to reported SNPs in miRNAs reported previously (Han and Zheng, 2013) and dbSNP v151. The identified miRNA M/E were also compared to miRNA editing sites reported in the RADAR (Ramaswami and Li, 2014) and DARNED (Kiran and Baranov, 2010) databases, and existing studies (Zheng et al., 2016; Lu et al., 2022). The results of these comparisons were listed in the last 6 columns in Supplementary Table S2.

### 2.5 PCA and clustering analysis using editing levels

We performed PCA analysis using the editing levels of editing sites identified in 448 single cell samples (as shown in Supplementary Table S8.3). The prcomp function in the psych package of R language was used to perform PCA analysis. The results of PCA were then visualized with MatLab (v2019) software (MathWorks, Natick, MA, US). The hclust function in the pheatmap package in R language was used to perform clustering analysis. The pheatmap function in the pheatmap package was used to visualize the result of the clustering analysis.

### 2.6 Identifying cell-type-specific M/E sites

To identify M/E sites that were specific to different cell type, we constructed 5 cell type vectors with 448 dimensions for 5 cell types, i.e., GBM (n = 165), HEK293FT (n = 66), Leuk (n = 19), pESC (n = 95) and nESC (n = 103), respectively. For example, the cell type vector for GBM has values of 1 for the 165 dimensions representing the 165 GBM samples, and 0 for other samples. Then, the correlation coefficient were calculated between the editing levels of 566 M/E sites in 448 samples and the 5 cell type vectors, respectively. An M/E site was regarded as specific to a specific cell type if the correlation coefficient between its editing levels and the cell type vector of this cell type is larger than 0.3 and P -value smaller than 0.05. We obtained 79, 31, 8, 30, and 78 M/E sites that were specific to GBM, HEK293T, Leuk, pESC and nESC, respectively (Supplementary Table S3).

### 2.7 Examining variations of editing levels of M/E sites in the same cell types

We first calculated the standard deviation (Std) of editing levels of 566 editing sites in 5 types of cells (as listed in Supplementary Table S4.1). Editing sites were then classified into 4 groups according to their Std values in the 5 types of cells: Std=0 , 0<Std≤0.2 , 0.2<Std≤0.3 , and 0.3<Std . We defined editing sites with Std smaller than or equal to 0.2 as low variance sites, Std between 0.2 and 0.3 (inclusively) as medium variance sites, and Std larger than 0.3 as high variance sites in a specific cell type. The numbers of M/E sites with low, medium and high variances in 5 types of cells were listed in Supplementary Table S4.2.

### 2.8 Identifying M/E sites with significantly different editing levels in different cell types

We compared editing levels of 566 M/E sites in GBM (n = 165), HEK293FT (n = 66), Leuk (n = 19), pESC (n = 95) with their editing levels in nESC (n = 103) with Mann-Whitney *U*-tests, respectively. The obtained *P*-values were corrected with the Benjamini–Hochberg correction method (Benjamini and Hochberg, 1995). M/E sites with *P*-values less than 0.05 were considered to have significant differences in the compared groups. The M/E sites with different editing levels in GBM, HEK293FT, Leuk and pESC compared to nESC were listed in Supplementary Table S5.

### 2.9 Identifying targets for original and edited miRNAs

We selected M/E miRNAs whose editing site occurs in the seed region of mature miRNAs with significantly different editing levels in GBM (n = 165), HEK293FT (n = 66), Leuk (n = 19), pESC (n = 95) when compared to nESC (n = 103). The targets of original and edited miRNAs were identified using the MiCPAR algorithm (Zheng, 2018) with its default parameters. In total, 11 PAR-CLIP sequencing profiles from two researches were downloaded from NCBI SRA database (as shown in Supplementary Table S1.2). The series accession number of one research is SRP002487, including seven stably expressing FLAG/HA -tagged *AGO1*, *AGO2*, *AGO3* and *AGO4* from HEK293 cells (Hafner et al., 2010). Another series accession number is SRP018015, which prepared HEK293 cell lines stably expressing *AGO* proteins labeled with HIS/FLAG/HA, and 3 expressed *AGO1* and 1 expressed *AGO2* (Memczak et al., 2013). The raw reads from 11 PAR-CLIP sequencing files were filtered and processed to obtain qualified reads. The remaining reads in these 11 profiles were combined and used in the identification of miRNA targets with the MiCPAR algorithm. The annotation of NCBI RefSeq genes in the GTF file, the mRNA sequences of NCBI RefSeq genes and soft-masked genome sequences of human (version hg38) were downloaded from the UCSC Genome Browser and used as inputs of the MiCPAR algorithm. The targets with at least 1 PAR-CLIP read with T-to-C variation were kept for further analysis. The MiCPAR algorithm used $P_{s}$ -value to measure the significance of the identified miRNA complementary site (Zheng, 2018).

To examine the expression of miRNA targets, four batches of public expression files were downloaded, including three batches of expression data for GBM and normal control tissue samples (CGGA (Zhao et al., 2021), GSE184643, and GSE205512), and one batch of single cell expression data of GBM and neural stem cells (NSC, GSE119834) (Marques et al., 2021).

### 2.10 GO terms and KEGG pathways enrichment analysis

The GO (Gene Ontology) terms and KEGG (Kyoto Encyclopedia of Genes and Genomes) pathway enrichment of the targets of the original and edited miRNAs were analyzed with KOBAS (v3) (Bu et al., 2021), respectively. The enriched GO terms were divided into three main categories, i.e., Biological Process, Cellular Component and Molecular Function. Then, the enriched GO terms and KEGG pathways of the original and edited miRNAs were compared. After comparing the targets of original and edited miRNAs, new targets of edited miRNAs were identified, and then KOBAS was used for GO term and KEGG pathway enrichment analysis of the new targets of edited miRNAs. Compared with nESC, editing levels of hsa-mir-376c_48_A_g increased in GBM cell samples. The target gene of hsa-mir-376c_48 g is expected to be downregulated in GBM samples. Therefore, the 568 new target genes of hsa-mir-376c_48 g predicted in this study were compared with the genes that were downregulated in published GBM samples (CGGA, GSE184643, GSE205512 and GSE119834). Totally, 76 target genes of hsa-mir-376a_48 g that were downregulated in at least one of the 4 public gene expression data sets selected were used to GO and KEGG analysis.

### 2.11 Correlation analysis between editing levels and expression of the enzyme genes

Because both the RNA-Seq and sRNA-Seq profiles were available for Leuk cells (n = 19), it was feasible to examine the potential correlation between the editing levels of the miRNA M/E sites and expression levels of their corresponding enzymes. The M/E sites were combined for the 19 Leuk cells (as listed in Supplementary Table S7). We then calculated the correlation between expression levels of *TENT* family members and editing levels of the 3′ end editing sites (as listed in Supplementary Table S6). The corrcoef function in MatLab (v2019, MathWorks, Natick, MA, US) was used to calculate the correlation values.

## 3 Results

### 3.1 Summary of M/E sites in single cells

We used the MiRME pipeline (Zheng et al., 2016) with default parameters to analyze 448 single-cell small RNA sequencing data. As shown in Supplementary Figure S1A and Supplementary Tables S2, S56 significant M/E sites were identified in the single-cell sRNA-seq profiles analyzed. We found that the percentages of 3′-U and 3′-A sites were the largest in the eight different categories of M/E sites identified in single-cell samples.

Next, according to the variation types of bases, the specific distribution of editing types of A-to-I and Other types sites were further investigated. 4 A-to-I editing events and 3 Other editing events were detected in 448 single-cell samples, and two deletion or insertion were also found (as shown in Supplementary Figure S1B; Supplementary Table S14.2).

Furthermore, the number of editing events occurring at the 5′, 3′ ends, and central regions of precursor miRNAs was counted. We found that editing events occurred more frequently at the 3′ end than at the 5′ end and in the central region. Only one or two editing events were observed at the 5′ end or central regions of pre-miRNAs (as shown in Supplementary Figure S1C; Supplementary Table S14.3), which was similar to the results reported previously based on bulk-seq data (Zheng et al., 2016; Wu et al., 2023).

As listed in Supplementary Table S2, the 566 significant M/E sites identified from the single-cell sRNA-Seq profiles were also compared to SNPs in miRNAs and reported editing sites in miRNAs (see Materials and Methods). All of the four A-to-I editing sites were reported previously (Supplementary Table S2). And most (85.5%) of the 3′ editing sites were also reported in at least one of the previous studies. These results suggested that miRNA M/E sites identified from single cell sRNA-Seq profiles were reliable.

### 3.2 Different cell types show different miRNA editing patterns

**FIGURE 1:**
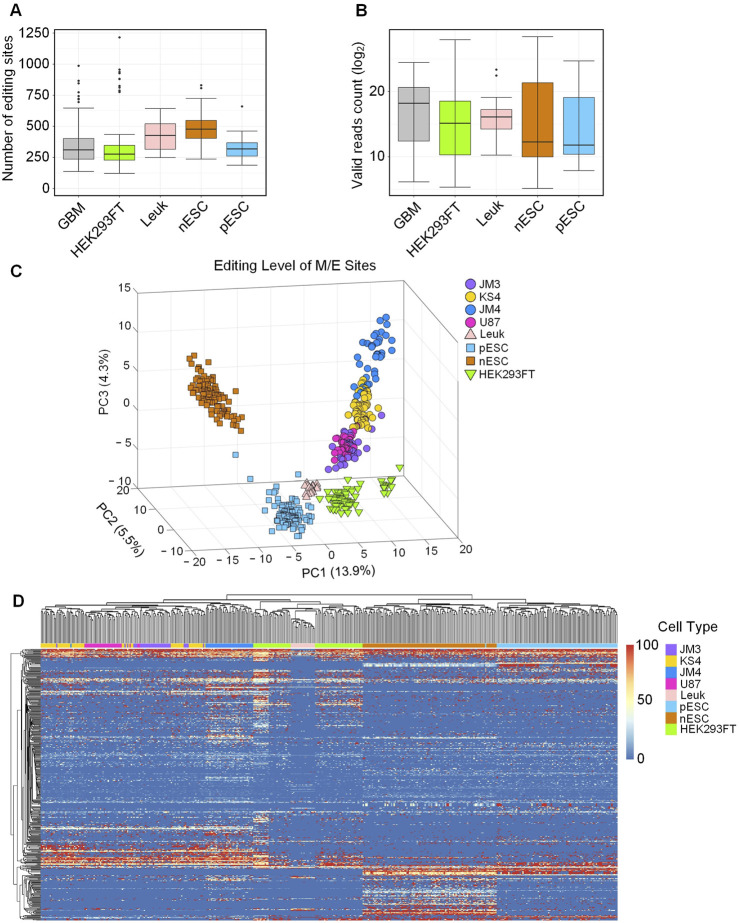
Different types of cells have different miRNA editing patterns. **(A)** The numbers of miRNA M/E sites identified in 5 types of cells. **(B)** The numbers of valid sequencing reads in 5 types of cells. **(C)** PCA using editing levels of 566 miRNA M/E sites. **(D)** Bi-clustering analysis using the editing levels of 566 miRNA M/E sites. In Part C to D, n (JM3) = 35, n (KS4) = 60, n (JM4) = 38, n (U87) = 32, n (Leuk) = 19, n (pESC) = 95, n (nESC) = 103, and n (HEK293FT) = 66. The source data are available in Supplementary Table S8.

To investigate whether there are differences in the number of miRNA editing sites among different cell types, we separately counted the number of editing sites identified in 448 samples. As shown in Figure 1A, different cell types had different number of M/E sites (as listed in Supplementary Table S8). Furthermore, nESC samples had highest median number of editing sites, while the largest number of editing events detected in a single sample was observed in HEK293FT samples, with 1,215 editing events. These results suggested that even for the same type of single cell, the number of miRNA M/E sites identified in different single cells was highly variable. As shown in Figures 1A,B, the valid reads in HEK293FT is relatively low (as shown in Supplementary Table S8.2), correspondingly, the number of M/E sites in HEK293FT is also small. This result suggests that the number of miRNA M/E sites detected within a single sample is affected by the number of valid reads or sequencing depth of that sample.

We performed PCA analysis on the editing levels of 566 miRNA M/E sites in the 448 single-cell samples. As shown in Figure 1C, nESC and pESC samples were clearly distinguished in the PCA analysis. The distance between pESC and HEK293FT samples was relatively close. This was consistent with previous results on miRNA expression pattern that there were significant differences in miRNA expression between nESC and pESC, and pESC and HEK293FT had more similar miRNA expression profiles (Faridani et al., 2016). The distances between nESCs, pESCs, and other cell types were relatively large, indicating substantial differences between ESCs and the other three differentiated cell types. The distance between undifferentiated nESC samples and other cell types was relatively large with clear boundaries, indicating that the miRNA editing pattern of nESCs was significantly different from those of other cell types. The 4 cell lines contained in GBM were gathered in a cluster. The boundary between JM3 and U87 was unclear, indicating that the miRNA editing patterns of the 2 cell lines were similar. Partial samples of JM4 and KS4 were mixed, and there were differences between them and JM3 and U87. In summary, our results indicated that samples from the same main cell types could be correctly clustered and the five main cell types could be clearly differentiated using the editing levels of miRNA M/E sites, suggesting that different types of cells had different miRNA editing patterns.

We next used the editing levels of the M/E sites to perform biclustering analysis. Results showed that nESC and pESC were clustered together while GBM, Leuk and HEK293FT were clustered together (Figure 1D), again suggesting that stem cells and differentiated cells had different miRNA editing patterns.

### 3.3 Cell-type specific M/E sites

**FIGURE 2:**
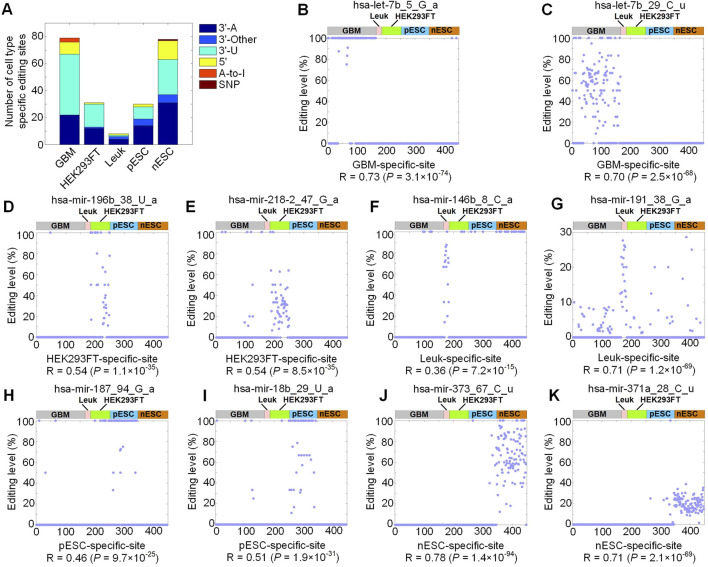
Cell-type specific miRNA M/E sites. **(A)** The numbers of cell-type specific M/E sites in different types of cells. Part **(B**–**K)** The editing levels of examples of cell-type specific M/E sites in the single cells selected. **(B)** hsa-let-7b_5_G_a, a GBM-specific site. **(C)** hsa-let-7b_5_G_a, a GBM-specific site. **(D)** hsa-mir-196b_38_U_a, an HEK293FT-specific site. **(E)** hsa-mir-218-2_47_G_a, an HEK293FT-specific site. **(F)** hsa-mir-146b_8_C_a, a Leuk-specific site. **(G)** hsa-mir-191_38_G_a, a Leuk-specific site. **(H)** hsa-mir-187_94_G_a, a pESC-specific site. **(I)** hsa-mir-18b_29_U_a, a pESC-specific site. **(J)** hsa-mir-373_67_C_u, an nESC-specific site. **(K)** hsa-mir-371a_28_C_u, an nESC-specific site. In all parts, n (GBM) = 165, n (Leuk) = 19, n (pESC) = 95, n (nESC) = 103, and n (HEK293FT) = 66. The source data are available in Supplementary Table S9.

Because different types of cells showed different miRNA editing patterns (Figures 1C,D), we hypothesized that there could be M/E sites happening in specific types of cells. By examining correlation between cell type feature vectors and editing levels of M/E sites (see Materials and Methods), we obtained 79, 31, 8, 30, and 78 M/E sites that were specific to GBM, HEK293FT, Leuk, pESC and nESC, respectively (Figure 2; Supplementary Table S6).

As shown in Figure 2A, most cell-type specific sites were 3′-U or 3′-A sites. In Figures 2B,C, these two sites defined as GBM-specific sites showed very high editing levels in GBM samples, but almost zero editing levels in other types of cells. Similar patterns were also noticed in other sites in Figures 2D–K. When comparing two sites in Figures 2H,I to two sites in Figures 2J,K, it could be seen that these sites were specifically edited in either pESC or nESC, providing a new source of the molecular differences between pESC and nESC.

### 3.4 M/E sites with different editing levels in same types of cells

To investigate whether the same M/E sites had same editing levels among different samples of the same type of cells, we calculated the standard deviations of each site in 5 types of samples. As shown in Supplementary Figure S2 and Supplementary Table S15, the distribution of standard deviation of 566 editing sites in 5 types of cells was different, ranging from 0.0 to 0.6. In 5 types of cells, i.e., GBM, Leuk, pESC, nESC and HEK293FT, there are 170, 51, 156, 147, 172 sites with high variations of editing levels, respectively (as shown in Supplementary Table S5).

**FIGURE 3:**
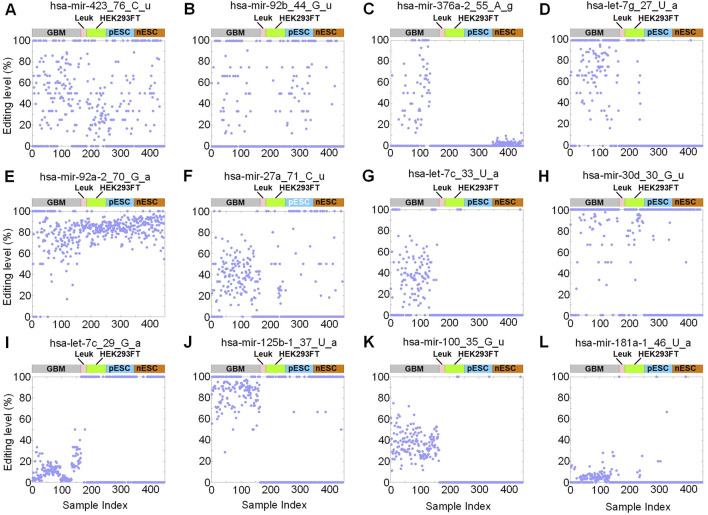
Selected M/E sites with different variances of editing levels in GBM samples. **(A-D)** Distributions of editing levels of 4 M/E sites with high variances. **(E-H)** Distributions of editing levels of 4 M/E sites with medium variances. **(I-L)** Distributions of editing levels of 4 M/E sites with low variances. In all parts, n (GBM) = 165, n (Leuk) = 19, n (pESC) = 95, n (nESC) = 103, and n (HEK293FT) = 66. The source data are available in Supplementary Table S10.

Then we selected 4 high, 4 medium, and 4 low variance sites in GBM respectively. As M/E sites with high variance in the GBM samples, the standard deviations of hsa-mir-423_76_C_u, hsa-mir-92b_44_G_u, hsa-mir-376a-2_55_A_g, hsa-let-7g_27_U_a were 0.34, 0.39, 0.33, 0.34, respectively (see Figures 3A–D; Supplementary Table S10). These sites showed large variations in the editing levels in the GBM samples. As M/E sites with medium variance in the GBM samples, the standard deviations of hsa-mir-92a-2_70_G_a, hsa-mir-27a_71_C_u, hsa-let-7c_33_U_a, hsa-mir-30d_30_G_u were 0.21, 0.21, 0.28, 0.29, respectively (see Figures 3E–H). These sites showed some variations in their editing levels in the GBM samples. The standard deviations of hsa-let-7c_29_G_a, hsa-mir-125b-1_37_U_a, hsa-mir-100_35_G_u, hsa-mir-181a-1_46_U_a were 0.10, 0.13, 0.12, 0.05, respectively (see Figures 3I–L). The editing levels of these sites were more uniformly located in a small range than those in Figures 3A–H.

As shown in Figure 3C, the editing levels of hsa-mir-376a-2_55_A_g was generally higher in GBM samples than that in nESC samples. The editing of this site was not detected in Leuk, HEK293FT and pESC samples.

### 3.5 M/E sites with different editing levels in differentiated cell types

To investigate the differences in editing levels of miRNA editing sites among different types of cell samples, highly undifferentiated nESC samples were used as controls to compare the editing levels of M/E sites in 4 differentiated cell types (i.e., GBM, Leuk, HEK293FT and pESC) with those in nESC (as shown in Supplementary Table S5).

**FIGURE 4:**
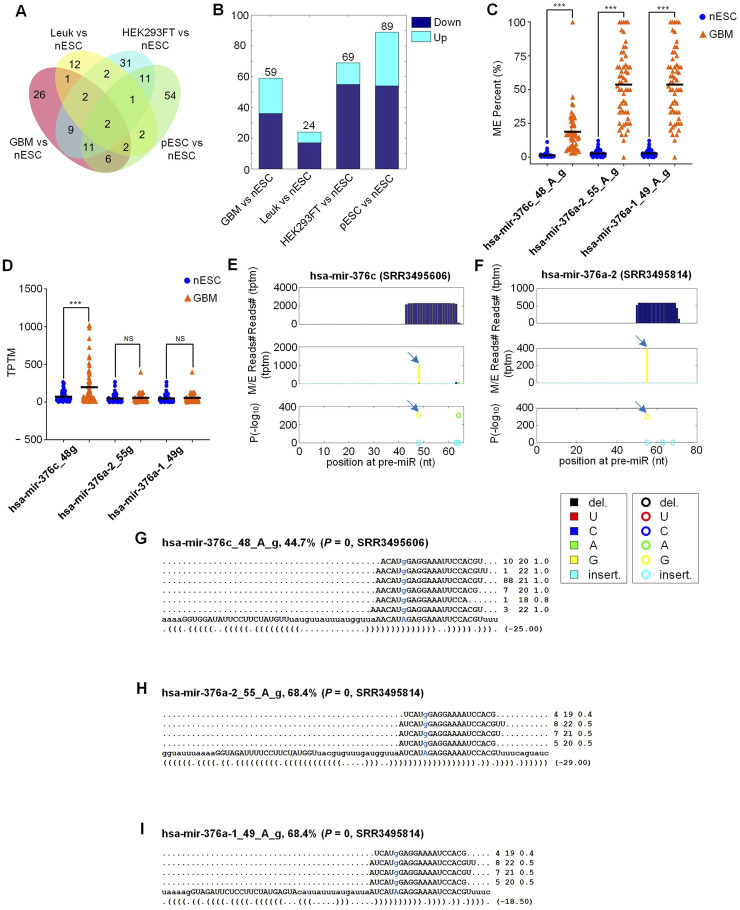
Comparing editing levels of M/E sites between nESC and 4 different cell types. **(A)** Overlap of sites with significant difference between 4 types of cells and nESC. **(B)** The change of editing levels. **(C)** Editing levels of 3 A-to-I sites in GBM (n (GBM) = 165) and nESC (n (pESC) = 95). **(D)** The abundances (TPTM, Tags Per Ten Million sequencing reads) of 3 A-to-I edited miRNAs corresponding to the three A-to-I editing sites in GBM (n (GBM) = 165) and nESC (n (pESC) = 95). **(E)** MiRME map of hsa-mir-376c in a glioblastoma cell (SRR3495606). **(F)** MiRME map of hsa-mir-376a-2 in a glioblastoma cell (SRR3495814). **(G)** The details of hsa-mir-376c_48_A_g in SRR3495606. **(H)** The details of hsa-mir-376a-2_55_A_g in SRR3495814. **(I)** The details of hsa-mir-376a-1_49_A_g in SRR3495814. In Part **(C,D)**, “***” indicates corrected *P*-values (with the Benjamini and Hochberg method (Benjamini and Hochberg, 1995)) smaller than 0.0001, “NS” indicates not significant, exact negative Binomial tests implemented in edgeR (Robinson et al., 2010). The source data are available in Supplementary Table S11.

As shown in Figure 4A and Supplementary Table S5, there were 59, 24, 69, and 89 M/E sites with significantly different editing levels when comparing GBM, Leuk, HEK293FT, and pESC with nESC, respectively. Only a few common sites with significantly different editing levels were found when comparing GBM, Leuk, HEK293FT, pESC with nESC (see Figure 4A), suggesting that the editing patterns in different cell types were very different which might contribute to their different differentiation pathways. In cells with higher differentiation, there tended to be more M/E sites with lower editing levels when compared to nESC cells (see Figure 4B).

We found that compared with nESC samples, three A-to-I editing sites, i.e., hsa-mir-376c_48_A_g, hsa-mir-376a-2_55_A_g, and hsa-mir-376a-1_49_A_g, had significantly different editing levels in two groups. The editing levels of three A-to-I editing sites are higher in GBM than in nESC (see Figure 4C; Supplementary Table S11). The expression levels of edited miRNA corresponding to hsa-mir-376c_48_A_g, i.e., hsa-mir-376c_48 g, was significantly upregulated in GBM compared with nESC (see Figure 4D). The detailed information of two A-to-I editing sites in hsa-mir-376c and hsa-mir-376a-2 in one of the GBM samples (SRR3495606 and SRR3495814, respectively) were presented in Figures 4E,F, respectively. The three A-to-I sites were central editng sites (see Figures 4G–I), and hsa-mir-376c_48_A_g located in the seed region of hsa-mir-376c-3p (Figure 4G).

### 3.6 Identifying targets of original and edited hsa-miR-376c-3p

To decrease false positive and increase the reliability of miRNA targets predicted in this study, 11 PAR-CLIP sequencing profiles were used. PAR-CLIP technology can isolate RNA fragments that bind to RNA binding proteins, enhancing the accuracy of predicting animal miRNA target genes (Hafner et al., 2010). We used MiCPAR algorithm (Zheng, 2018) to identify the targets of original and edited miRNAs by analyzing 11 PAR-CLIP profiles of AGO proteins. The results showed that the original and edited hsa-mir-376c-3p had 107 common target genes, and hsa-mir-376c_48 g obtained 568 new target genes (see Supplementary Figure S3; Supplementary Table S16).

**FIGURE 5:**
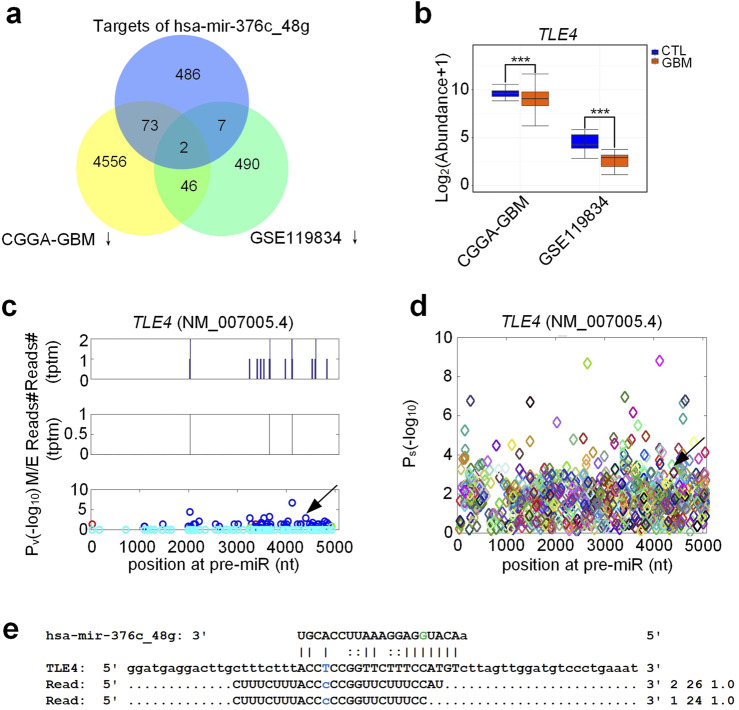
Target analysis of A-to-I edited hsa-mir-376c-3p, i.e., hsa-mir-376c_48g. **(a)** Overlap of new target genes of hsa-mir-376c_48g and genes downregulated in GBM in two batches of public data. **(b)** The expression of *TLE4* in GBM and normal control (CTL) in two batches of public data. For the CGGA-GBM data, the numbers of GBM and CTL samples are 140 and 20, respectively. For GSE119834, the numbers of GBM and CTL samples are 45 and 9, respectively. **(c)** Distribution of PAR-CLIP reads on *TLE4* (NM_007005.4). **(d)** The identified miRNA sites and their $P_{s}$ values on *TLE4*. **(e)** The details of complementary sites of hsa-mir-376c_48g and PAR-CLIP reads on *TLE4*. “***” represent *P*-value less than 0.0001, exact negative Binomial tests implemented in edgeR. The source data are available in Supplementary Table S12.

Next, we compared the new target genes of hsa-mir-376c_48 g to downregulated genes in GBM. We found that in the two gene expression data sets selected (CGGA and GSE119834), 2 target genes of hsa-mir-376c_48 g were significantly downregulated in GBM (see Figure 5a; Supplementary Table S12). As shown in Figure 5b, the expression level of *TLE4* in GBM samples was significantly lower than that in normal control samples, which is consistent with the increased expression of hsa-mir-376c_48 g in GBM samples (Figure 4D). Figure 5c showed the distribution of PAR-CLIP reads for *TLE4*, and result indicated that PAR-CLIP reads were significantly accumulated at the complementary sites of hsa-mir-376c_48G. The identified miRNA complementary sites and their $P_{s}$ value on *TLE4* were shown in Figure 5d. Figure 5e showed the details of complementary sites of hsa-mir-376c_48 g on *TLE4* (NM_007005.4), as well as the PAR-CLIP reads at these sites.

Besides, we obtained three batches of public expression data of GBM and normal tissues. We compared the 568 new target genes of hsa-mir-376c_48 g to deregulated genes in GBM tissues. We found that in three sets of gene expression profiles selected (CGGA, GSE184643 and GSE205512), 4 target genes of hsa-mir-376c_48 g were significantly downregulated in GBM (see Supplementary Figure S4A; Supplementary Table S17). Among 4 genes, *RIMS3* (Regulating Synaptic Membrane Exocytosis 3) has the function of ensuring the binding activity of transmembrane transporters and participates in multiple biochemical and physiological processes, including neurotransmitter exocytosis regulated by calcium ions, regulation of chemical synaptic transmission, and regulation of synaptic structures.

As shown in Supplementary Figure S4B,C, in three batches of public data (CGGA, GSE184643 and GSE205512), the expression levels of *RIMS3* and *ATP1B1* in GBM samples were significantly downregulated compared to normal control samples, respectively. As shown in Supplementary Figure S4D,E, many PAR-CLIP reads were accumulated at the complementary sites of hsa-mir-376c_48 g on *RIMS3* and *ATP1B1*, respectively. The identified complementary sites of hsa-mir-376c_48 g and their $P_{s}$ values on *RIMS3* and *ATP1B1* were shown in Supplementary Figure S4F,G, respectively. As shown in Supplementary Figure S4H,I, some PAR-CLIP reads carrying T-to-C variation were accumulated on complementary sites of hsa-mir-376c_48 g on *RIMS3* (NM_014747.2) and *ATP1B1* (NM_001677.3), respectively.

### 3.7 GO terms and KEGG pathways enrichment analysis

To investigate the function of target genes of hsa-mir-376c_48 g, we performed GO and KEGG analysis on 76 genes that were predicted as target genes of hsa-mir-376c_48 g and were downregulated in at least one of the four public gene expression data sets selected (see Materials and methods). As shown in Supplementary Figure S5A and Supplementary Table S18, the 76 genes were mainly enriched in GO terms such as “fatty acid elongation”, “ATP metabolic process”, “positive regulation of neurotrophin TRK receptor signaling pathway”, “cytosol”, “protein binding” and “ATP binding”.

As shown in Supplementary Figure S5B, these 76 genes were enriched in KEGG pathways such as “Metabolic pathways”, “Phagosome”, “Endocytosis”, “Choline metabolism in cancer”, “Neurotrophin signaling pathway” and “Synaptic vesicle cycle”. These results suggested that the 76 target genes of hsa-mir-376c_48 g were involved in the maintenance of normal function of the nervous system.

### 3.8 Correlation analysis between enzymes and editing levels

**FIGURE 6:**
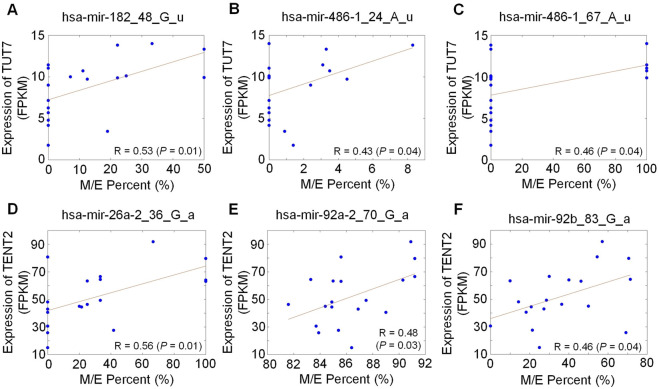
The correlations between expression levels of *TENT* genes and editing levels of selected 3′ editing sites in the 19 Leuk samples. **(A)** The correlation between expression of *TUT7* and editing level of hsa-mir-182_48_G_u. **(B)** The correlation between expression of *TUT7* and editing level of hsa-mir-486_1_24_A_u. **(C)** The correlation between expression of *TUT7* and editing level of hsa-mir-486_1_57_A_u. **(D)** The correlation between expression of *TENT2* and editing level of hsa-mir-26a_2_36_G_a. **(E)** The correlation between expression of *TENT2* and editing level of hsa-mir-92a_2_70_G_a. **(F)** The correlation between expression of *TENT2* and editing level of hsa-mir-92b_83_G_a. In all parts, n (Leuk) = 19. *P*-values were calculated based on the *t*-tests. The source data are available in Supplementary Table S13.

Because the RNA sequencing profiles were available for the 19 Leuk cells, we calculated the correlations between expression levels of *TENT* genes and editing levels of 3′ editing sites (as shown in Supplementary Table S6). We found 60 3′-A editing sites whose editing levels were significantly positively correlated with expression levels of one of the *TENT* family members (see Supplementary Table S6.1). For examples, as shown in Figures 6A–C, the expression levels of *TENT2* were positively correlation with three 3′-A editing sites, i.e., hsa-mir-26a-2_36_G_a (R = 0.56, *P* = 0.01), hsa-mir-92a-2_70_G_a (R = 0.48, *P* = 0.03), hsa-mir-92b_83_G_a (R = 0.46, *P* = 0.04). We found twenty six 3′-U editing sites whose editing levels were significantly positively correlated with expression levels of *TENT* family members (see Supplementary Table S6.2). As shown in Figures 6D–F, the expression levels of *TUT7* were positively correlated with 3 3′-U editing sites, i.e., hsa-mir-182_48_G_u (R = 0.53, *P* = 0.01), hsa-mir-486-1_24_A_u (R = 0.43, *P* = 0.04), hsa-mir-486-1_57_A_u (R = 0.46, *P* = 0.04). These results indicate that the three 3′-A editing sites, i.e., hsa-mir-26a-2_36_G_a, hsa-mir-92a-2_70_G_a and hsa-mir-92b_83_G_a, may be mediated by *TENT2*, and the three 3′-U editing sites, i.e., hsa-mir-182_48_G_u, hsa-mir-486-1_24_A_u, hsa-mir-486-1_57_A_u, may be mediated by *TUT7*.

Additionally, we found twenty eight 3′-C editing sites whose editing levels were significantly positively correlated with expression levels of *TENT* family members (see Supplementary Table S6.3; Supplementary Figure S6). The expression levels of *TENT2* were significantly positively correlated with the editing levels of hsa-mir-21_31_U_c (see Supplementary Figure S6A; Supplementary Table S19). The expression levels of *TUT1* were significantly positively correlated with the editing levels of hsa-mir-148a_65_U_c (see Supplementary Figure S6B). The expression levels of *TENT4A* were significantly positively correlated with the editing levels of hsa-mir-26a-1_32_G_c (see Supplementary Figure S6C). The expression levels of *TENT4B* were significantly positively correlated with the editing levels of hsa-mir-374b_31_U_c (see Supplementary Figure S6D). The expression levels of *TENT5B* were significantly positively correlated with the editing levels of hsa-mir-374b_31_U_c (see Supplementary Figure S6E). The expression levels of *TENT5B* were significantly positively correlated with the editing levels of hsa-mir-486-2_22_G_c (see Supplementary Figure S6F). These results suggest that *TENT2*, *TENT4A*, *TENT4B*, *TENT5A*, *TENT5B*, *TENT5C*, *TUT1* and *TUT4* potentially involved in modification of some miRNA molecules by adding a cytosine to their 3′ ends.

We also found that the expression level of *TENT5A* was significantly positively correlated with the editing levels of hsa-mir-320a_70_A_g (see Supplementary Figure S7). This result suggested that *TENT5A* might contribute to 3′ guanylation on some miRNAs.

Meanwhile, the 3′ end editing of some miRNAs, may jointly be mediated by different TENT, enzymes. As shown in Supplementary Figure S8A and Supplementary Tables S21, S3′-A editing of 4 miRNAs, i.e., hsa-mir-10b-5p, hsa-mir-23a-3p, hsa-mir-197-3p and hsa-mir-454-3p, were collaboratively mediated by TENT4A and TENT5C. similarly, 3′-C editing of hsa-mir-99a-5p may be jointly mediated by TENT4A and TENT5C.

As shown in Table 1, we summarized the possible nucleotides that might be added to the 3′ ends of miRNAs by different members of the *TENT* enzyme family.

**TABLE 1:** The 3′ nucleotide addition events of miRNAs that are mediated by the *TENT* family.

*TENT* member	Adenine	Uracil	Cytosine	Guanine
*TENT2*	Y*	Y*	Y	*
*TENT4A*	Y	Y	Y	N
*TENT4B*	Y*	Y	Y	N
*TENT5A*	Y	N	Y	Y
*TENT5B*	Y	Y	Y	N
*TENT5C*	Y	Y	Y	N
*TENT5D*	Y	N	N	N
*TUT1 (TENT1)*	Y	Y	Y	N
*TUT4 (TENT3A)*	N	*	Y	N
*TUT7 (TENT3B)*	Y	Y*	N	N

Y indicates that the mediation relationship is supported by the results of this study, N indicates that the mediation relationship is not supported by the results of this study, and * indicates that the mediation relationship has been reported by existing literature (Wyman et al., 2011; Yu and Kim, 2020; Yang et al., 2022; Boele et al., 2014; Jones et al., 2009; Yang et al., 2019).

## 4 Discussion

In this study, we used MiRME algorithm to identify the miRNA M/E sites in 448 single-cell sRNA sequencing profiles, and 566 M/E sites were identified. In single cells, 3′ editing events of miRNAs account for the largest portion among different types of editing events. Our results show that 3′-A and 3′-U are more prevalent than other 3′ editing events. These results were similar to those noticed from bulk sRNA-Seq profiles (Zheng et al., 2014; 2016; Wang Q. et al., 2019; Guo et al., 2022; Xie et al., 2022; Lu et al., 2022; Wu et al., 2023; Liu et al., 2023).

Our results showed that miRNA editing patterns were different in five types of single cells, i.e., GBM, Leuk, nESC, pESC and HEK293FT. We found that the some miRNA M/E sites show large variances in the editing levels in different single cells of the same cell type. And some miRNA M/E sites exhibit relatively uniform editing levels in cell samples of the same cell type.

We found that an A-to-I site, hsa-mir-376c_48_A_g, had significantly higher editing levels and expression levels in GBM compared to nESCs. We analyzed target genes of original/edited hsa-mir-376c-3p with the MiCPAR algorithm, and found that hsa-mir-376c_48 g obtained 568 new target genes. We hypothesized that hsa-mir-376c_48 g might be involved in the occurrence and progression of GBM by potentially repressing the expression of *RIMS3*, *ATP1B1* and *TLE4*. Further experimental studies are needed to verify the direct target relationship between hsa-mir-376c_48 g and *RIMS3*, *ATP1B1* and *TLE4*. Because the sample sizes of different cell types are limited and the sample sizes of different cell types are different, more experiments are necessary to further validate the abnormal editing of hsa-mir-376c-3p.

*TLE4* is significantly upregulated within chronic rapamycin-treated glioblastoma multiforme (GBM) cells, and is a key modulator of resistance to mammalian target of rapamycin (mTOR) pathway specific inhibition (Laks et al., 2012). Wang et al. (2021) revealed that targeted knockout of the *TLE4* gene enhanced the efficacy of CAR in the therapeutic pathway for GBM. *TLE4* was reported as a tumor suppressor and when the *TLE4* gene was deleted, leukemia cells gained proliferative activity (Shin et al., 2016). Lin et al. reported that reducing the expression of *TLE4* in papillary thyroid carcinoma could lead to the proliferation, migration and invasion of cancer cells, and activate the JAK/STAT pathway, while overexpression of *TLE4* could inhibit the growth and metastasis of cancer cells (Lin et al., 2024).

The roles of *ATP1B1* and *RIMS3* in GBM are still not clearly clarified. In primary GBM, the expression level of *ATP1B1* in astrocytes was very low, even close to the state of no expression (Rotoli et al., 2017). The protein encoded by *ATP1B1* belongs to the $Na^{+}/K^{+}$ and $H^{+}/K^{+}$ ATPase β chain protein family and the $Na^{+}/K^{+}$ ATPases subfamily. It was reported that gliomas could utilize ion channels and transporters such as ATPase to maintain their rapid growth and invasion ability, allowing them to invade brain tissues (Skou, 1998). *RIMS3* could serve as a key gene for predicting the prognosis of colorectal cancer patients (Deng et al., 2022). Low expression of *RIMS3* was associated with poor prognosis in CRC patients, and the expression level of *RIMS3* was significantly lower in tumor samples compared to normal samples (Deng et al., 2022).

As summarized in Table 1, previous studies reported that *TENT2* could mediate adenylation, uridylation and guanylation at the 3′ end of specific miRNA molecules (Yang et al., 2022; Wyman et al., 2011). It was reported that *TENT2* mediated the monoadenylation of mature miRNA molecules, including miR-31, let-7a, let-7d, let-7 g, let-7b, and miR-92a, in HCT-116 cell line (Wyman et al., 2011). *TENT2* catalyzed the monoadenylation of mature miRNA molecules let-7d, let-7i, miR-122, miR-145, and miR-98, increasing their stability (D’Ambrogio et al., 2012). *TENT2* also mediated the monoadenylation of miR-26a and miR-27a, thereby reducing the inhibition of target mRNA (Burroughs et al., 2010). *TENT4B* mediated the monoadenylation of let-7a, miR-15a, miR-100, and miR-200c (Wyman et al., 2011). *TENT4B* mediated the monoadenylation of miR-21 and promoted the degradation of miRNA molecules (Boele et al., 2014). *TUT4* catalyzed the monouridylation of miR-26a-5p, reducing the inhibition of target mRNA (Jones et al., 2009). *TUT4* and *TUT7* catalyzed the monouridylation of miR-27a (Yang et al., 2019).

In addition to the confirmed enzyme activities mentioned above, our results suggested that other members of the *TENT* enzyme family, including *TENT4A*, *TENT5A*, *TENT5B*, *TENT5C*, *TENT5D*, and *TUT1*, might also mediate nucleotide addition events at the 3′ end of miRNAs (as listed in Table 1). The 3′-A editing of hsa-mir-454-3p, hsa-mir-197-3p, hsa-mir-10b-5p and hsa-mir-23a-3p, and the 3′-C editing of hsa-mir-99a-5p, may be mediated simultaneously by *TENT4A* and *TENT5C*. Therefore, our results also suggest that some *TENTs* might competitively involve in the 3′-end editing of some miRNAs. However, due to the limitation of sample size, more investigations are needed to clarify the mechanisms of *TENT* enzymes and 3′ end editing.

## 5 Conclusion

Previous studies reported many miRNA editing sites of different types based on bulk sRNA-seq profiles, some of which had abnormal editing in diseases. Here, we reported 566 miRNA M/E sites after analyzing 448 single-cell sRNA sequencing profiles with the MiRME algorithm. Our results showed that the editing patterns of miRNAs were different in different types of cells, which indicated that the heterogeneity of cells may be reflected according to their miRNA editing patterns. Our results also revealed that the same M/E sites might have very different editing levels in different individual cells of the same cell types, suggesting advantages of single cell sRNA-Seq profiles over bulk sequencing ones in detecting the largely neglected differences within individual single cells even from the same cell types. We hypothesize that hsa-mir-376c_48 g might play a role in the pathophysiology of GBM, by potentially repressing *RIMS3*, *ATP1B1* and *TLE4*. Furthermore, our results suggested that more members of the *TENT* enzyme family possessed broader capabilities to mediate 3′-editing of miRNAs than those reported in existing studies.

## Data Availability

The original contributions presented in the study are included in the article/Supplementary Material, further inquiries can be directed to the corresponding author.
