# Supplementary material for: Characterizing miRNA editing patterns in 5 types of cells using single-cell small RNA sequencing data
Source: Front Bioinform. 2026 Apr 9;6:1719535. doi: 10.3389/fbinf.2026.1719535 (PMC13102661; doi:10.3389/fbinf.2026.1719535)
Supplement: Supplementary file 1 [file DataSheet1.pdf]

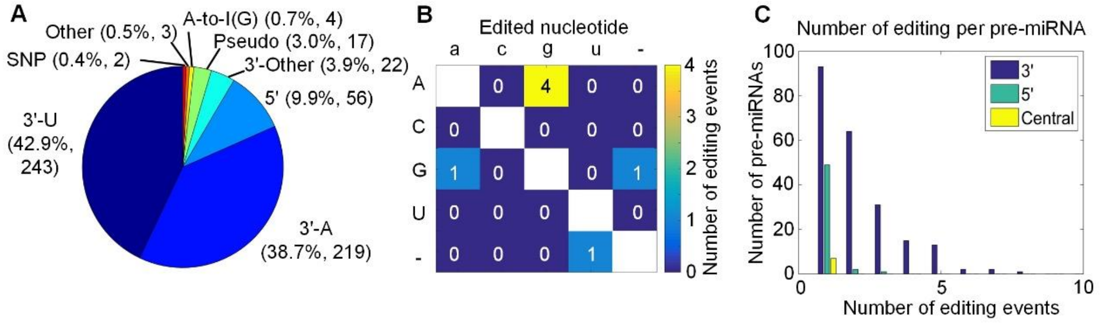

Figure S1. The summary of miRNA M/E sites in 448 single cell small RNA sequencing samples. (A) Summary of 566 miRNA M/E sites. (B) Distribution of 7 central miRNA M/E sites. (C) Frequency of miRNA M/E events and corresponding pre-miRNA quantity. The source data are available in Table S14.

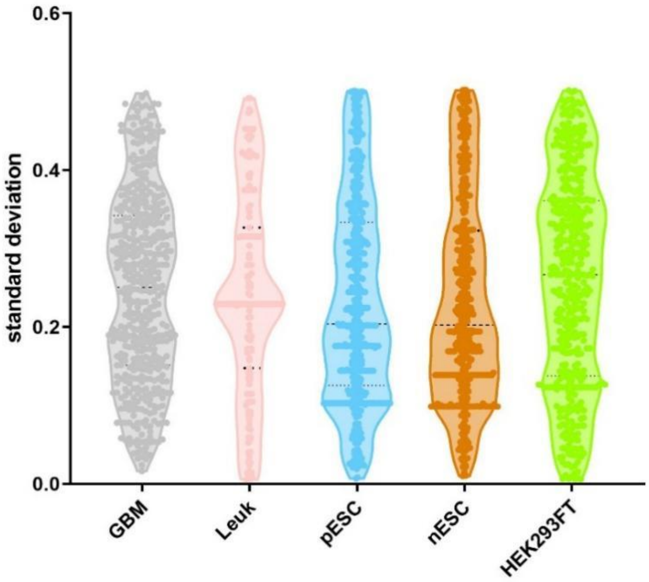

Figure S2. The distribution of standard deviation of 566 editing sites in 5 types of cells. The source data are available in Table S15.

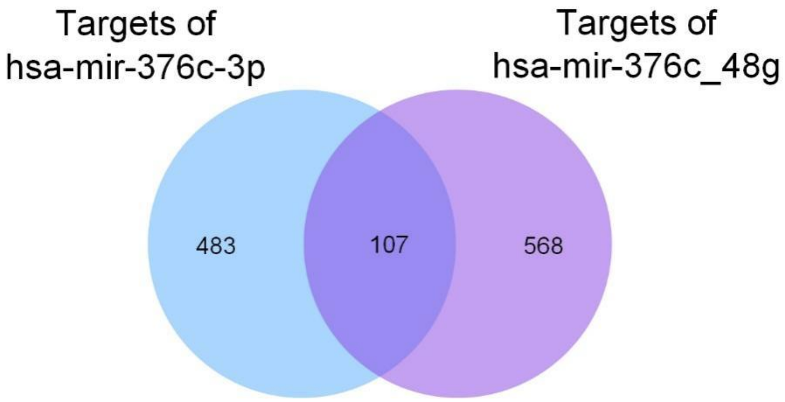

Figure S3. Comparisons of the targets of hsa-mir-376c-3p and that of hsa-mir-376c_48g. The source data are available in Table S16.

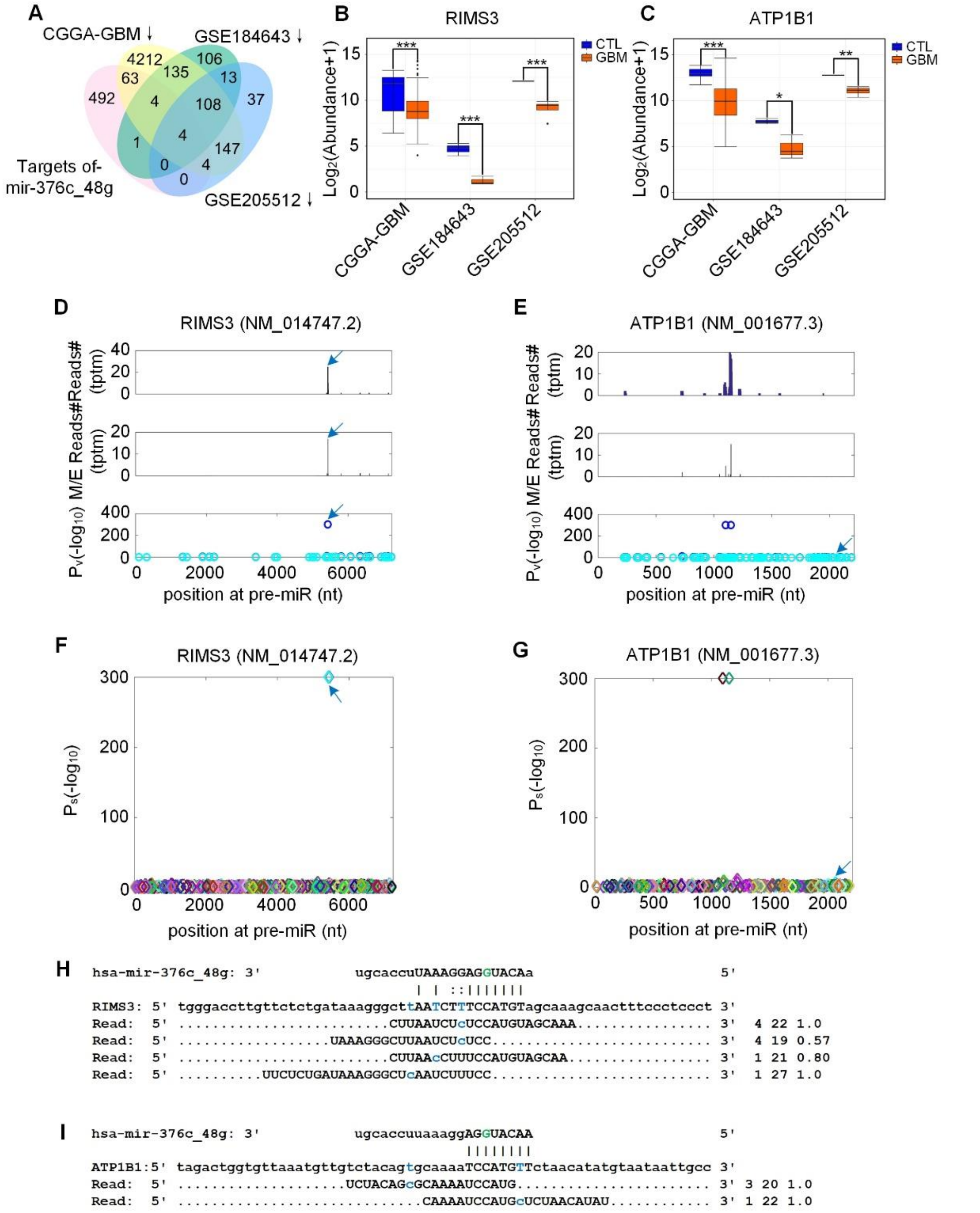

Figure S4. Additional targets of hsa-mir-376c_48g. (A) The overlap between new target genes of hsa-mir-376c_48g and genes down-regulated in GBM in three batches of public data. (B) The expression of *RIMS3* in GBM and normal control (CTL) in three batches of public data. (C) The expression of *ATP1B1* in GBM and CTL in three batches of public data. (D) Distribution of PAR-CLIP reads on *RIMS3* (NM_014747.2). (E) Distribution of PAR-CLIP reads on *ATP1B1* (NM_001677.3). (F) The identified miRNA sites and their *P*s values on *RIMS3*. (G) The identified miRNA sites and their *P*s values on *ATP1B1*. (H) The details of complementary sites of hsa-mir-376c_48g and PAR-CLIP reads on *RIMS3*. (I) The details of complementary sites of hsa-mir-376c_48g and PAR-CLIP reads on *ATP1B1*. In Part B and C, "***", "**", and "*" represent *P*-values less than 0.0001, 0.001 and 0.01 (edgeR), respectively. The source data are available in Table S17.

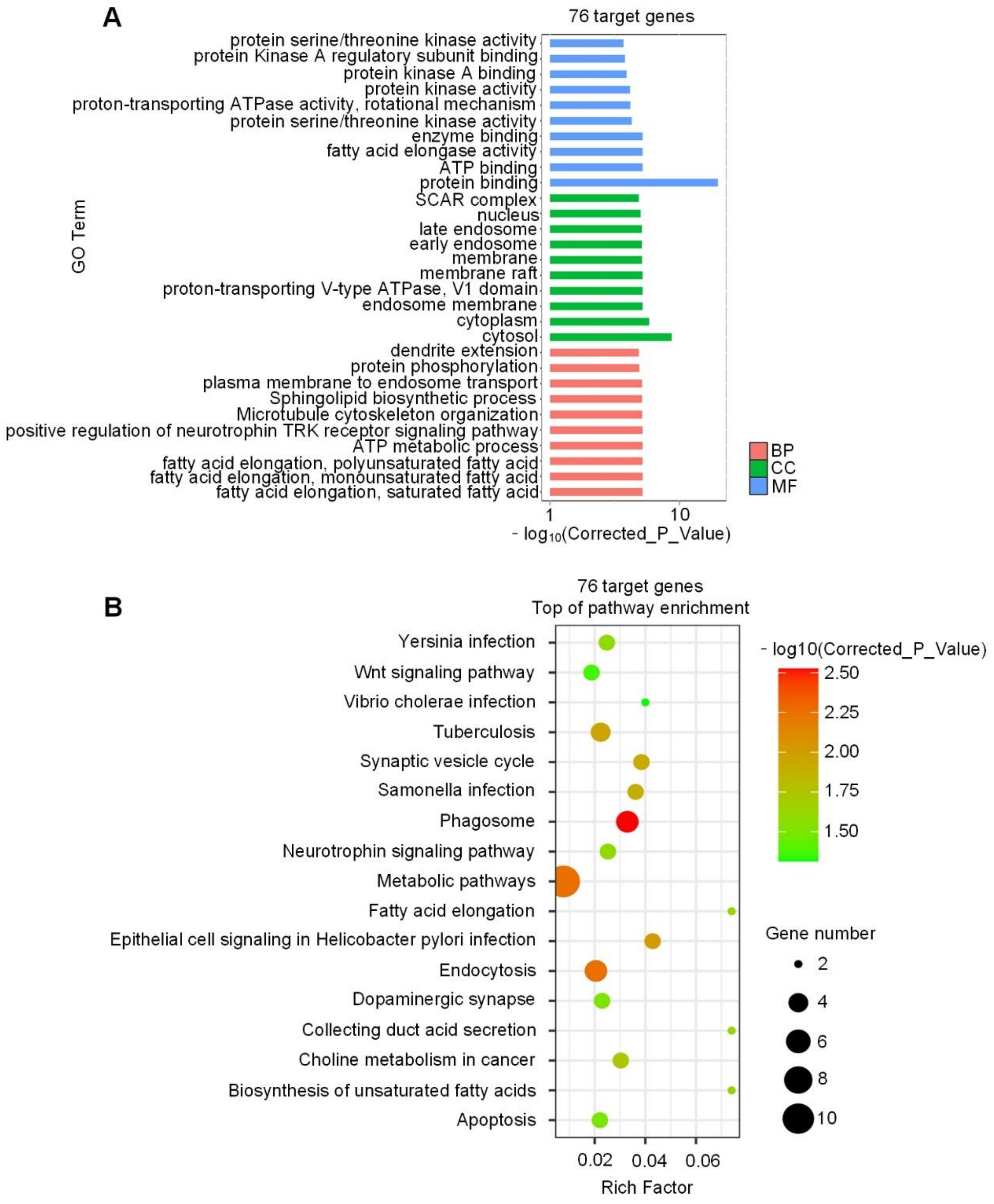

Figure S5. The enriched GO terms and KEGG pathways of 76 target genes of hsa-mir-376c_48g. (A) Enriched GO terms of 76 genes. (B) Enriched KEGG pathways of 76 genes. The source data are available in Table S18.

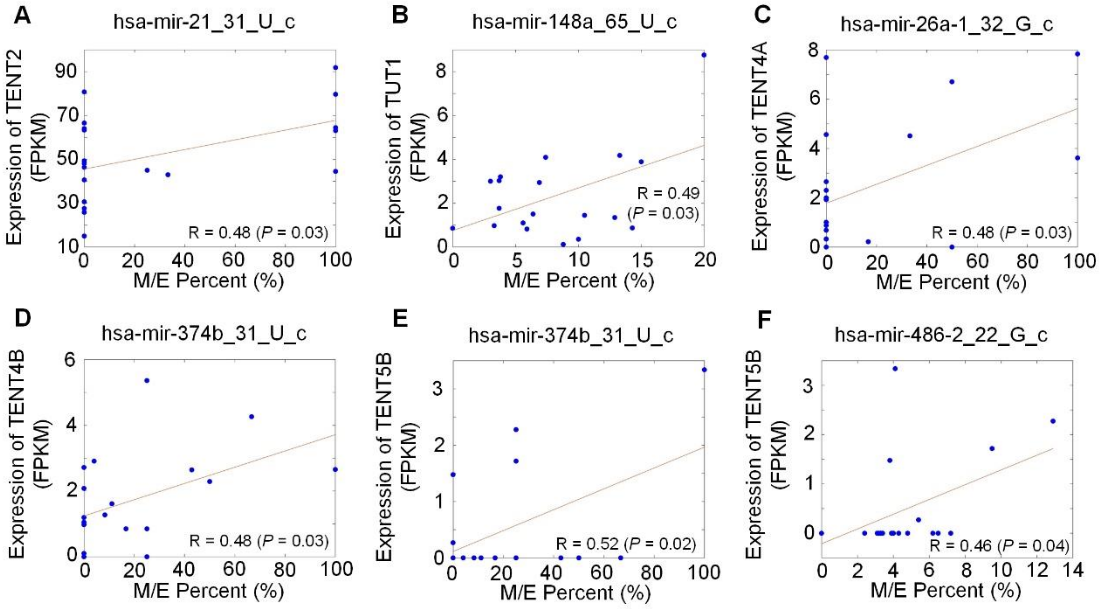

Figure S6. The correlation between *TENT* family members and 3'-C editing sites (A) The correlation between expression of *TENT2* and editing levels of hsa-mir-21_31_U_c. (B) The correlation between expression of *TUT1* and editing levels of hsa-mir-148a_65_U_c. (C) The correlation between expression of *TENT4A* and editing levels of hsa-mir-26a-1_32_G_c. (D) The correlation between expression of *TENT4B* and editing levels of hsa-mir-374b_31_U_c. (E) The correlation between expression of *TENT5B* and editing levels of hsa-mir-374b_31_U_c. (F) The correlation between expression of *TENT5B* and editing levels of hsa-mir-486-2_22_G_c. *P*-values were calculated based on *t*-tests. The source data are available in Table S19.

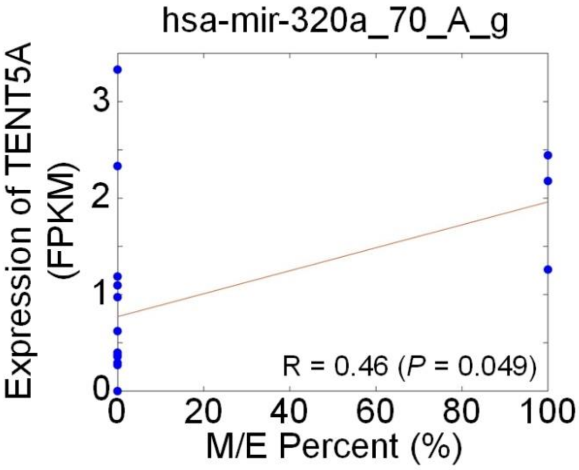

Figure S7. The correlation between expression of *TENT5A* and editing levels of hsa-mir-320a_70_A_g. *P*-value was calculated based on *t*-test. The source data are available in Table S20.

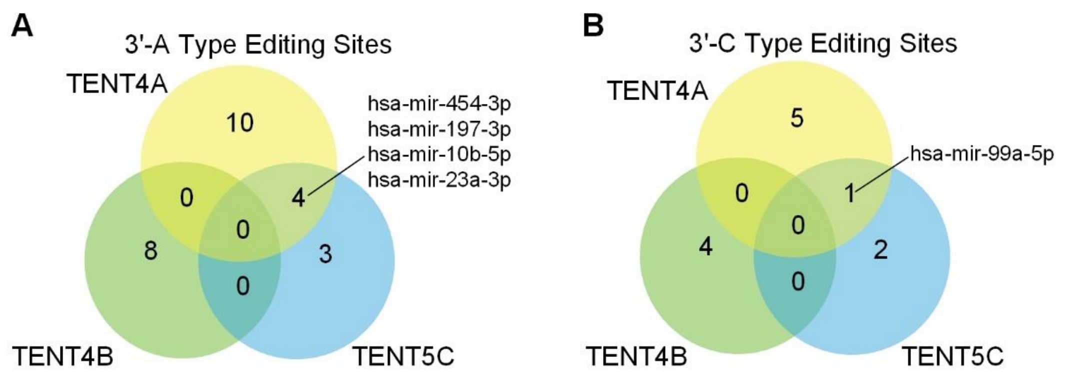

Figure S8. The miRNAs whose 3' editing events may be mediated by different TENT family members. (A) Overlap of miRNAs whose 3'-A editing events are potentially mediated by TENT4A, TENT4B, and TENT5C. (B) Overlap of miRNAs whose 3'-C editing events are potentially mediated by TENT4A, TENT4B, and TENT5C. The source data are available in Table S21.
